# Mendelian Randomization Identifies Lipidomic Signatures of Depression Risk That Are Partly Reflected in Cortisol-Induced Membrane Remodeling and Modulated by St. John’s Wort Extract (Ze 117)

**DOI:** 10.3390/ijms27104344

**Published:** 2026-05-13

**Authors:** Virginie Freytag, Veronika Butterweck, Dominique J.-F. de Quervain, Georg Boonen, Andreas Papassotiropoulos

**Affiliations:** 1 Division of Molecular Neuroscience, Department of Biomedicine, University of Basel, CH-4055 Basel, Switzerland; andreas.papas@unibas.ch; 2 Research Cluster Molecular and Cognitive Neurosciences, Department of Biomedicine, University of Basel, CH-4055 Basel, Switzerland; dominique.dequervain@unibas.ch; 3 Psychiatric University Clinics, University of Basel, CH-4055 Basel, Switzerland; 4 Max Zeller Soehne AG, Medical Department, CH-8590 Romanshorn, Switzerland; veronika.butterweck@zellerag.ch (V.B.); georg.boonen@zellerag.ch (G.B.); 5 Division of Cognitive Neuroscience, Department of Biomedicine, University of Basel, CH-4055 Basel, Switzerland

**Keywords:** depression, St. John’s wort, Ze 117, lipidomics, Mendelian randomization, cholesteryl ester

## Abstract

Major depressive disorder (MDD) is associated with altered membrane lipids, but the causal species remain uncertain. Using two-sample Mendelian randomization (MR) on lipidomic GWAS data and the latest MDD meta-analysis (~400,000 cases; 1.5 million controls), we identified 49 lipid species linked to MDD risk, notably enriched for phosphatidylcholines. Protective lipids were enriched for long-chain polyunsaturated fatty acids (20:3–20:5), whereas shorter-chain or less unsaturated species, particularly 18:2-containing lipids, increased risk. These associations were also observed in a subset of clinically assessed MDD cases. Colocalization supported shared causal variants between many lipid traits and MDD, prominently at the *FADS1/2* locus and additional loci, suggesting multiple entry points into lipid metabolism that differ partly from bipolar disorder. MR-implicated lipid shifts overlapped with cortisol-induced changes in a human cell stress model and were often reversed by co-treatment with St. John’s wort extract (Ze 117). Cholesteryl ester 20:3 emerged as a robust candidate marker, showing protective MR effects in two cohorts, colocalizing genetic support, normalization by Ze 117, and an inverse correlation with depressive symptom severity in a non-clinical sample. Together, these results define a depression-associated lipidomic signature centered on polyunsaturated fatty acid metabolism with biomarker and therapeutic potential.

## 1. Introduction

Altered membrane lipid composition has repeatedly been linked to mood disorders [1] and to the pharmacodynamic action of certain antidepressants [2]. In particular, a deficiency in long-chain polyunsaturated fatty acids (PUFAs), especially omega-3 PUFAs, has been observed in patients with depression, alongside a relative overrepresentation of pro-inflammatory omega-6 fatty acids [3,4,5,6]. However, it remains unclear which specific lipid species lie on the causal pathway to major depressive disorder (MDD) and whether these lipid abnormalities are a cause or consequence of the disease. Recent metabolomic and genetic studies have begun to address this question. For example, Mendelian randomization (MR) has identified an imbalance in polyunsaturated fatty acids—such as lower docosahexaenoic acid and a higher omega-6/omega-3 ratio—as potentially causal in MDD [6]. Another MR study reported that higher eicosapentaenoic acid (20:5, an omega-3 PUFA) and adrenic acid (a downstream omega-6 PUFA) are protective against depression, whereas shorter-chain precursors (e.g., alpha-linolenic acid, 18:3 *n*-3) may increase depression risk [7]. These findings support a role for fatty acid metabolism in the etiology of depression, yet they were limited to a relatively small subset of metabolites or fatty acids. A comprehensive screen of the lipidome is needed to pinpoint the exact lipid species driving MDD risk.

In addition, it remains unknown whether the lipid perturbations linked to MDD might be pharmacologically reversible—and if so, whether effective antidepressant treatments act in part by normalizing these lipid profiles. Certain antidepressants are known to interact with membrane lipids; for instance, chronic treatment with selective serotonin reuptake inhibitors can remodel lipid raft microdomains and redistribute G protein signaling components in the membrane [2]. St. John’s wort extract (including Ze 117), a plant-derived antidepressant with efficacy in mild-to-moderate depression [8,9,10], has been shown to induce membrane lipid changes in vitro [11,12]. Importantly, data from a human cell-based stress model demonstrated that cortisol treatment led to pronounced reductions in membrane lipid levels and increased membrane fluidity, effects that were partially reversed by co-treatment with Ze 117 [13]. Recent in vivo experiments revealed that Ze 117 alters the plasma and hippocampal lipidome in stressed rats, particularly by increasing lysophosphatidylethanolamines (LPEs) and modulating lipid saturation and chain length [14]. However, despite these mutually reinforcing in vitro and in vivo data, it remains unclear whether the lipid changes induced by cortisol or Ze 117 are related to the specific lipid alterations observed in MDD.

To address this question, we aimed to integrate genetic epidemiology and experimental pharmacology to identify lipidomic signatures with a genetically inferred causal link to MDD and to determine their overlap with lipid changes induced by cortisol and Ze 117. First, we performed a two-sample MR analysis using large-scale genome-wide association study (GWAS) datasets to systematically screen blood lipid species for causal effects on MDD risk. We then tested the robustness of these findings via sensitivity MR methods, colocalization analyses (to ensure that lipid–MDD associations share the same causal variants), and utilization of additional independent lipidomics cohorts. We also performed a secondary MR in which variants within the pleiotropic *FADS* (fatty acid desaturase) locus were masked, allowing us to (i) determine whether lipid–MDD associations persist independently of this dominant cluster and (ii) contrast the resulting *FADS*-independent lipidomic signature of MDD with that observed for bipolar disorder. Finally, leveraging data from the aforementioned in vitro model of cortisol-induced lipid perturbation, we examined whether the lipid species identified as causal in MDD are also altered by cortisol and normalized by Ze 117. Additionally, we evaluated in a non-clinical cohort whether one top candidate lipid was associated with depressive symptom severity. Through this multifaceted approach, we sought to characterize a genetically informed lipidomic profile of depression and assess its potential therapeutic relevance, thereby clarifying the role of lipid metabolism in MDD pathogenesis and, possibly, treatment.

## 2. Results

### 2.1. Mendelian Randomization Identifies Genetic Components of the Lipidome Linked to MDD

**Figure 1:**
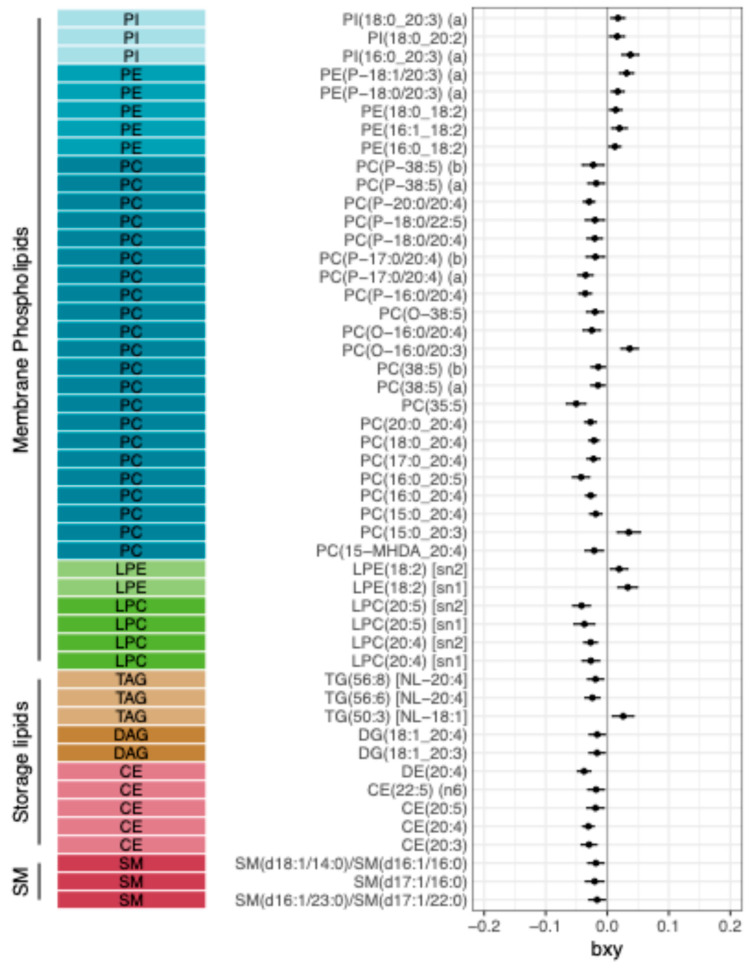
GSMR results between lipid species and MDD. List of 49 individual lipid species showing significant GSMR estimates (FDR < 0.05) with MDD. Error bars correspond to 95% confidence intervals. bxy: GSMR beta coefficient as log-odds of the outcome per 1 SD in the exposure. CE: cholesteryl ester; DAG: diacylglycerol; DE: dehydrocholesteryl ester; LPC: lysophosphatidylcholine; LPE: lysophosphatidylethanolamine; PC: phosphatidylcholine; PE: phosphatidylethanolamine; PI: phosphatidylinositol; SM: sphingomyelin; TAG: triacyglycerol.

We performed a comprehensive two-sample MR investigation of the genetic link between the blood lipidome and MDD. Using the Cadby et al. lipid GWAS panel (578 lipid species; *n* = 6057 individuals) [15] and the largest available GWAS for MDD (≈412 k cases, 1.59 M controls) [16], GSMR identified 49 individual lipid species significantly associated with risk of developing MDD (false discovery rate *q* < 0.05). These results are summarized in Figure 1 and Supplementary Table S1. Notably, sensitivity analyses yielded consistent effect estimates for a majority of these hits across alternative MR methods (IVW and MR-Egger; see Supplementary Table S1 and Figure S1), indicating that the findings are robust to potential pleiotropy.

The contribution of subclinical cases in the large MDD GWAS (which included many self-reported or questionnaire-ascertained cases from the UK Biobank) raised the question of clinical relevance. We therefore repeated the GSMR analysis using a stricter, clinically ascertained MDD GWAS (26,778 cases based on clinical interview, 51,857 controls). This analysis identified 26 lipid species significantly associated with clinically diagnosed MDD (*q* < 0.05; Supplementary Table S2 and Figure S2), of which 20 overlapped with the 49 hits from the full sample (extended definition). Thus, lipid associations discovered in the larger community-based sample were also evident in clinically defined MDD, underscoring their relevance to diagnosable depressive illness.

The 49 lipid species linked to MDD span multiple lipid classes, but there was a striking overrepresentation of phosphatidylcholines (PCs). Nearly half (45%) of the MDD-associated lipids were PCs, despite PCs comprising only ~19% of all species tested (Supplementary Table S1; Fisher’s exact test *p* = 0.009). Notably, total blood PC concentration itself was not significantly associated with MDD (GSMR estimate = −0.0064, *p* = 0.44; Supplementary Table S1), suggesting that the enrichment of PCs among the hits is not driven by a global shift in PC levels, but rather by specific PC species. Examining fatty acid chain characteristics, we observed a clear pattern: lipid species containing shorter-chain unsaturated fatty acids (particularly linoleic acid, 18:2) were associated with increased risk of MDD, whereas species containing longer-chain unsaturated fatty acids (e.g., arachidonic acid 20:4; eicosapentaenoic acid 20:5; docosapentaenoic acid 22:5) were associated with lower risk of MDD. This pattern is illustrated in Figure 1. An interesting exception was observed for lipids containing the 20:3 fatty acid. In membrane lipids (e.g., plasmalogen PC(O-16:0/20:3)), the 20:3 acyl chain was associated with higher MDD risk, whereas in storage lipids (e.g., diacylglycerol DG(18:1/20:3) and cholesteryl ester CE(20:3)), higher levels were associated with lower MDD risk (protective effect). These opposing directions suggest that the biochemical context of the 20:3 fatty acid—whether it is incorporated into membrane structural lipids versus stored/transport lipids—may influence its relationship with depression. In addition to the glycerolipids, we identified three sphingomyelin (SM) species significantly associated with MDD, all of which showed protective directions (higher SM associated with lower MDD risk) (Figure 1).

We next sought to replicate and extend these findings using independent lipid GWAS datasets. Applying GSMR to the Ottensmann et al. lipid panel (179 species in a Finnish cohort) [17], we identified 18 lipid species significantly associated with MDD risk (*q* < 0.05; Supplementary Table S3 and Figure S3). Importantly, six (33%) of these overlapped with the 49 discovery hits from the Cadby panel, with consistent directions of effect: PI(18:0/20:3), PC(18:0/20:4), PC(17:0/20:4), PC(16:0/20:4), LPE(18:2), and CE(20:4). Notably, one sphingomyelin species (SM 40:2; O2) was identified in the Finnish sample, mirroring the negative (protective) association with MDD observed for sphingomyelins in the discovery analysis. In the lipid panel from Harshfield et al. [18], we found 14 lipid measures significantly associated with MDD (*q* < 0.05; Supplementary Table S3 and Figure S3). Three (21%) of these corresponded to our discovery hits—namely CE(20:3), CE(20:5), and LPC(20:4)—all with consistent protective directions. Furthermore, two additional sphingomyelins in the Harshfield data showed negative genetic associations with MDD, congruent with our findings.

Across the 49 discovery lipid hits, 20 were also mapped by the Ottensmann panel; of those, 18 (90%) showed a concordant direction of effect in the Finnish sample, and eight (40%) were nominally significant (*p* < 0.05) for an MDD association (Figure S4). Likewise, 10 of the 49 hits were also mapped by the Harshfield panel, of which six had nominally significant, concordant associations (Figure S4). These observations reinforce the validity of the identified lipid–MDD links.

Expanding our view beyond dedicated lipid panels, we analyzed a metabolome-wide dataset (Metabolon platform; 726 metabolites, *n* = 8455 individuals) [19]. Using GSMR, we identified 38 metabolites significantly associated with MDD risk (*q* < 0.05; Supplementary Table S4 and Figure S5). The majority of these metabolites were lipids or lipid-related molecules, consistent with the lipidome constituting a major portion of the metabolome. Strikingly, the same fatty acid pattern emerged: long-chain PUFA-containing lipids (e.g., 20:4 and 20:5 species) were generally associated with lower MDD risk, whereas shorter-chain unsaturated lipids (notably those containing 18:2) were associated with higher risk (Figure S5). For instance, two metabolites containing 20:3 fatty acyl chains (annotated as 1-stearoyl-2-meadonanoyl-GPC (18:0/20:3*n*-9) and 1-dihomo-γ-linolenoyl-GPE (20:3*n*-3 or *n*-6)) showed protective associations with MDD in the metabolome analysis. These observations align with the lipidomic findings and suggest that perturbations in fatty acid elongation/desaturation pathways are robustly implicated in depression risk.

In summary, our MR results provide evidence for a widespread genetic link between MDD and the lipidomic fraction of the metabolome. The pattern of effects—with omega-6 and omega-3 long-chain PUFAs generally conferring protection and their shorter precursors conferring risk—was consistent across multiple cohorts and platforms. This consistency points to a coherent underlying biological signature rather than spurious findings.

### 2.2. Colocalization Analysis Highlights Multiple Genomic Loci Shared Between Lipid Traits and MDD

To determine whether the lipid-associated genetic signals genuinely reflect shared causal variants with MDD (as opposed to merely co-occurring signals in the same regions), we performed colocalization analyses. Across the genome, we identified six loci with strong evidence of a shared causal variant between at least one lipid trait and MDD (colocalization posterior PPH4 > 0.8), and an additional 12 loci with suggestive evidence of colocalization (PPH4 > 0.5) (see Supplementary Table S5 for full results). The most prominent colocalization signals clustered on chromosome 11 within a ~1 Mb region encompassing the *FADS1* and *FADS2* genes (the fatty acid desaturase cluster). This locus harbors pleiotropic variants known to affect the blood levels of numerous fatty acids and lipids, and it appears to drive a substantial fraction of the lipid–MDD associations. In our analysis, 44 of the 49 MR-implicated lipid species had at least one associated SNP in the FADS region, and many showed colocalization with MDD at this locus. Fine-mapping of the colocalized region pointed not only to *FADS2* (the Δ6-desaturase) as a likely mediator, but also to nearby genes such as *TMEM258*, *MYRF*, and *FEN1*, which were represented among the 95% credible set variants (Supplementary Table S5). Notably, using the Ottensmann et al. lipid data, we also observed colocalization at the FADS locus with MDD (Supplementary Table S6), underscoring the robustness of this shared genetic architecture across cohorts.

**Figure 2:**
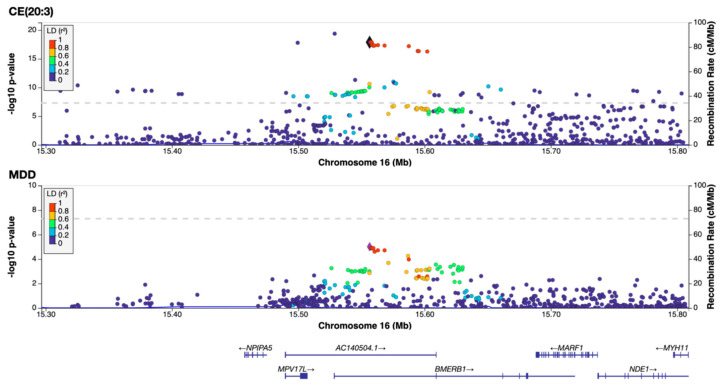
Visualization of colocalizing region between CE(20:3) and MDD on chromosome 16. GWAS association signals for CE(20:3) and MDD within chromosome 16 locus showing strong evidence of shared causal variant between both traits (PPH4 > 0.8). LD reference SNP corresponds to the SNP exhibiting highest posterior probability of colocalization within the locus.

These findings are in line with previous reports linking mood disorders to fatty acid desaturase variants and PUFA levels [6,7]. Beyond the *FADS* region, our colocalization analysis highlighted several additional loci. For example, we found evidence of a shared causal variant (PPH4 > 0.8) at the *BMERB1* locus (chromosome 16) for a lipid trait (CE(20:3)) and MDD, pointing to a gene involved in the regulation of microtubules and cerebral cortex development (Figure 2). Interestingly, this genomic locus also exhibited a strong colocalization signal between MDD and two additional 20:3 fatty acid-containing lipid measures obtained using the Metabolon platform (1-oleoyl-2-dihomo-linolenoyl-GPC 18:1/20:3 and 1-stearoyl-2-dihomo-linolenoyl-GPC 18:0/20:3 *n*-3 or *n*-6) (Supplementary Table S7). Other loci of interest included regions on several chromosomes, each harboring lipid-associated variants that colocalized with MDD signals (PPH4 > 0.5). While these non-FADS loci individually affected fewer lipid traits, they suggest that multiple independent biological pathways connect lipid metabolism to depression. Detailed locus-specific results are provided in Supplementary Tables S5–S7.

In summary, the colocalization analysis supports a shared genetic basis for many of the lipid associations with MDD. The canonical *FADS1/2* locus emerges as a central hub—consistent with its key role in PUFA biosynthesis—but the presence of additional colocalized loci indicates that the genetic link between the lipidome and MDD is not confined to a single pathway.

### 2.3. Differential Contribution of the FADS Gene Cluster in MDD vs. Bipolar Disorder

Given the strong colocalization signal between MDD and lipid traits at the *FADS* gene cluster, we repeated the GSMR analysis after excluding this locus (chr11:61,067,099–62,134,286). This exclusion caused a substantial drop in association signals, leaving only two lipid species meeting the significance threshold (FDR *q* < 0.05): cholesteryl ester CE(20:3) and plasmalogen phosphatidylcholine PC(P-16:0/20:4) (Supplementary Table S8). However, out of the 49 lipid species originally linked to MDD, 11 still showed nominal associations with MDD (with consistent effect directions) even after removing the *FADS* locus (Figure S6). This suggests the existence of *FADS*-independent metabolic mechanisms contributing to MDD, notably involving certain sphingomyelin and phospholipid species. A similar pattern was observed in other lipidomic and metabolomic datasets when applying the same *FADS* locus exclusion (Figure S6).

The *FADS* gene cluster is strongly linked to the risk for bipolar disorder (BD) [20]. We therefore performed a parallel GSMR analysis for BD, leveraging the largest GWAS to date (59,287 cases and 781,022 controls) [21]. When including the *FADS* region, GSMR identified 61 circulating lipid species significantly associated with BD risk (FDR *q* < 0.05; Supplementary Table S9). Notably, 41 of these overlapped with the 49 MDD-linked lipids, and the GSMR effect estimates for lipid species were highly concordant between MDD and BD (Pearson *r* ≈ 0.8 across 168 species tested in both), with generally larger effect sizes observed for BD (Figure S7). Upon excluding the *FADS* locus, however, only a single lipid species (CE(18:0)) remained significantly associated with BD (FDR *q* < 0.05; Supplementary Table S10). Only an additional seven lipids showed nominal associations with BD under *FADS* exclusion (all with consistent directions of effect; Supplementary Table S10), highlighting the dominant role of the *FADS* gene cluster in driving the lipid–BD genetic signal. Furthermore, without the *FADS* locus, the correspondence between MDD and BD GSMR estimates dropped markedly (correlation *r* ≈ 0.46), indicating that beyond the shared *FADS*-related effects, there may be partly distinct lipidomic components influencing MDD and BD. This finding points to the possibility of disorder-specific membrane lipid remodeling processes, although larger BD GWAS may eventually uncover additional *FADS*-independent lipid associations for BD.

### 2.4. Cholesteryl Ester 20:3 Levels Are Linked to Depressive Symptoms in Healthy Young Adults

Our genetic analyses converged on cholesteryl ester 20:3 (CE(20:3)) as a particularly intriguing lipid species consistently showing a protective association with MDD. In the primary GSMR analysis, higher genetically predicted blood levels of CE(20:3) were significantly associated with reduced depression risk, and this association remained robust after multiple-testing correction. Notably, CE(20:3) also retained significance in the clinically ascertained MDD subsample, underscoring its potential relevance to diagnosable depressive illness (Figure 1; Supplementary Tables S1 and S2). It also showed significant association with MDD in the Harshfield et al. lipid panel (Figure S3). Moreover, CE(20:3) was implicated in two independent colocalized loci with MDD (on chromosomes 11 and 16, Supplementary Table S5), strengthening the evidence of its association with MDD. These lines of evidence prompted us to examine whether CE(20:3) levels correlate with depressive symptoms in an independent sample of individuals.

We measured CE(20:3) in a cohort of *n* = 970 healthy young adults (mean age ~22 years) who underwent blood lipidomic profiling and completed the Montgomery–Åsberg Depression Rating Scale (MADRS) as a measure of depressive symptoms (see Methods). Consistent with the MR-predicted direction, we found that individuals with higher CE(20:3) levels had significantly lower depressive symptom scores (*r* = –0.079, *p* = 0.015; Figure S8). To ensure that this was not merely reflecting a general effect of cholesterol levels on mood, we calculated the fraction of CE(20:3) relative to total cholesteryl esters in each sample. This ratio (CE(20:3)/total CE) also showed an inverse association with MADRS scores (*r* = –0.07, *p* = 0.034), suggesting that it is the specific 20:3 content in the cholesteryl ester pool, rather than overall cholesterol, that relates to depressive symptoms.

We further constructed a polygenic score for CE(20:3) levels using the top SNP instruments from the GSMR. This genetic score was strongly associated with measured CE(20:3) concentrations in our young adult cohort (*r* = 0.15, *p* = 1.4 × 10^−6^), validating that the genetic predictors of this lipid are capturing actual variation in the trait. However, the CE(20:3) polygenic score was not significantly associated with MADRS scores in this sample (*r* = –0.02, *p* = 0.43), which is not surprising given the limited power and the small effect size linking this lipid to depression. Thus, while the genetic score did not directly predict depressive symptoms (precluding a mediation test), the observational association between CE(20:3) and mood in this cohort aligns with our MR findings. Together, these results highlight CE(20:3) as a potential biomarker linking lipid metabolism to depression, meriting further investigation.

### 2.5. Lipidomic Signatures of MDD Overlap with Cortisol-Induced Membrane Remodeling

One proposed mechanism in depression pathology is hyperactivation of the hypothalamic–pituitary–adrenal (HPA) axis, leading to elevated cortisol levels that can alter brain lipid metabolism [22]. We recently characterized the lipidomic effects of cortisol in a PBMC model and found that cortisol exposure induced a broad reduction in lipid levels, consistent with altered membrane fluidity and increased cellular stress [13]. Given the genetic evidence implicating altered levels of many lipids as risk factors for MDD, we examined the overlap between the MDD-associated lipidome and the cortisol-affected lipidome.

In the PBMC model, cortisol treatment (1 µM, 4 days) significantly downregulated 132 lipid species relative to untreated controls (FDR *q* < 0.05). Among those, 47 species were mapped by at least one lipid species investigated in Cadby et al., from which 11 overlapped with the 49 GSMR hits identified as significantly related to MDD (Supplementary Table S11). This overlap was significant (permutation *p* < 0.005, see Section 4). Moreover, nine of these eleven overlapping lipids had a negative GSMR effect estimate (i.e., lower lipid levels associated with higher MDD risk), aligning with the fact that cortisol reduced those lipid levels. In other words, the pattern of lipid changes induced by cortisol in cells—a broad lipid depletion—mirrors the direction of lipid changes that genetically increase MDD risk. This convergence suggests that cortisol-induced stress may promote depression in part by inducing a lipidomic profile that resembles the one causally linked to MDD by genetics.

Next, we investigated the effects of Ze 117 (St. John’s wort extract) on the cortisol-altered lipidome. Co-treatment with Ze 117 (50 µg/mL) in the cortisol-exposed PBMCs produced significant changes in 238 lipid species compared to cortisol alone (FDR *q* < 0.05 for Ze 117 effect). Notably, Ze 117 partially reversed the cortisol-induced lipid deficits: of the 132 lipids downregulated by cortisol, 75 (56.8%) were significantly changed in the opposite direction by Ze 117 (i.e., increased in the presence of Ze 117; see Supplementary Table S11). This indicates a broad normalizing or compensatory effect of the extract on the cellular lipidome under stress conditions.

Among the identified species, 79 were also included in the Cadby et al. lipidome GWAS and 12 were part of the 49 GSMR hits identified as genetically linked to MDD (Supplementary Table S11). While the overlap between Ze 117-regulated lipids and GSMR hits did not reach statistical significance in permutation testing (*p* > 0.05), the trend was noteworthy—especially considering that many lipids showed changes in the expected direction (i.e., Ze 117 increasing levels of lipids that are protective in MDD).

One noticeable example is CE(20:3), which was significantly increased by Ze 117 co-treatment relative to cortisol alone (log_2_ fold-change +1.55, *p* < 6.2 × 10^−3^) in the PBMC experiment. This aligns with our genetic and observational findings that higher CE(20:3) is associated with lower depression risk and fewer symptoms. Similarly, multiple long-chain PC species that were reduced by cortisol (and associated with MDD risk in MR) showed partial restoration towards baseline with Ze 117. These results, taken together, provide proof of concept that the lipidomic perturbations genetically linked to depression can be pharmacologically targeted and reversed, at least in a cellular model.

## 3. Discussion

In this study, we identified a distinctive lipidomic signature that shows a statistically inferred causal relationship with depression and demonstrated that this signature converges with lipid changes observed in a human cell-based stress model and normalized by the antidepressant Ze 117. Using MR on large-scale GWAS data, we found that individuals genetically predisposed to have lower levels of certain lipids—particularly those incorporating long-chain polyunsaturated fatty acids—are at higher risk for MDD. Conversely, genetic proxies for higher levels of long-chain PUFA-containing lipids (notably those with 20:3, 20:4, or 20:5 fatty acyl chains) were protective against depression. These findings extend and refine prior metabolomic studies that implicated PUFAs in depression [6,7], by pinpointing the specific lipid species (e.g., certain phosphatidylcholine, sphingomyelin, and cholesteryl ester molecules) that drive this association. They also align with epidemiological observations that diets rich in omega-3 fatty acids are associated with lower depression risk, whereas higher omega-6 intake or an elevated omega-6/omega-3 ratio is associated with increased risk [3,4,5,6]. Indeed, randomized trials and meta-analyses have shown that omega-3 PUFA supplementation yields a modest antidepressant effect [23], lending support to the idea that enhancing certain lipid levels can improve mood.

A central player in the lipid–depression link emerging from our analysis is the fatty acid desaturase (*FADS*) gene cluster on chromosome 11. This locus encodes the Δ5- and Δ6-desaturase enzymes that convert shorter PUFAs (like linoleic acid 18:2 and α-linolenic acid 18:3) into longer, more unsaturated PUFAs (such as arachidonic acid 20:4 and eicosapentaenoic acid 20:5). We found that genetic variation in the *FADS* region underlies a substantial portion of the lipidomic differences associated with MDD: essentially, alleles that decrease the activity of these desaturases (and thereby lower long-chain PUFA levels while raising short-chain precursors) were associated with higher depression risk. This result aligns with two recent genetic studies: one in depression, which identified polyunsaturated fatty acid dysregulation as potentially causal [6], and one in bipolar disorder, which highlighted *FADS*-driven arachidonic acid synthesis as a key factor [20]. Interestingly, Stacey et al. (2024) found that higher arachidonic acid levels (reflecting more active desaturase conversion) were associated with lower risk of bipolar disorder, and that no such association was detectable for MDD in their dataset [20]. Our findings suggest that with a much larger MDD sample, the same biological signal can be captured for depression: increased flux through the FADS pathway—yielding more long-chain omega-3 and omega-6 PUFAs—appears protective against MDD, whereas a bottleneck at this pathway (resulting in accumulation of shorter, less unsaturated lipids like 18:2) increases risk. Together, these genetic convergences point to perturbations in membrane PUFA composition as a shared pathophysiology across mood disorders, while also hinting at potential differences in magnitude or specificity between MDD and BD that merit further exploration.

Beyond the *FADS* genes, our colocalization analysis and *FADS*-masked MR highlighted additional loci that differentiate MDD from BD, suggesting disorder-specific membrane remodeling. These loci include regions harboring genes not classically associated with lipid biology, suggesting novel mechanistic insights. For instance, the locus near *BMERB1* (encoding BMERB domain-containing protein 1, also known as C16orf45) that was identified in our colocalization analysis may represent a new regulatory gene influencing lipid profiles—a hypothesis that warrants functional follow-up. Interestingly, a recent GWAS of red blood cell fatty acids identified a locus near *BMERB1* associated with altered membrane lipid composition. In that study, variants in the *BMERB1* region showed genome-wide significance for levels of polyunsaturated fatty acids (linoleic acid and dihomo-γ-linolenic acid (DGLA)) in red blood cell membranes [24]. To summarize, the overlap between MDD and BD lipid associations at the *FADS* locus suggests that an important part of the observed signal reflects a shared mood disorder biology centered on PUFA metabolism, and possibly broader metabolic processes, rather than a biomarker signature specific for MDD. At the same time, the persistence of a smaller set of *FADS*-independent associations in MDD, together with the reduced MDD-BD correspondence after *FADS* exclusion, leaves open the possibility of more disorder-enriched lipid components. Accordingly, the identified lipids should at present be viewed primarily as indicators of underlying pathophysiology or treatment-related lipid remodeling.

Our integrative approach also demonstrates how genetic findings can be connected to pharmacological mechanisms. Some genetically protective lipids were increased by Ze 117 in a cellular stress context, suggesting that membrane lipid remodeling is not only a correlate of depression but also a viable therapeutic target. Ze 117 (a low-hyperforin *Hypericum perforatum* extract) is known to have antidepressant efficacy comparable to standard antidepressants in mild-to-moderate MDD [9,10], and mechanistic studies have shown that it can influence membrane properties [11,12,13]. Its low hyperforin content (≤0.2%) minimizes the risk of pharmacokinetic drug interactions, particularly those mediated via cytochrome P450 enzymes and transporters, thereby offering a favorable safety profile compared to high-hyperforin extracts [25]. In an experiment utilizing human peripheral blood mononuclear cells (PBMCs), Ze 117 broadly counteracted cortisol-induced lipid depletion, effectively shifting the cellular lipidome toward a state that the genetic data indicate is protective (i.e., higher levels of key lipids) [13]. This convergence supports the concept that part of the therapeutic action of certain antidepressants—including plant-based ones like Ze 117—may involve normalizing aberrant lipid metabolism linked to stress. It is tempting to speculate that other antidepressant treatments, such as SSRIs, might similarly impact lipid profiles in beneficial ways. In fact, chronic SSRI treatment has been associated with increases in specific membrane lipids and restoration of lipid raft organization in preclinical studies [2]. Our results encourage further research into this membrane lipid hypothesis of antidepressant action. If particular lipid changes are a common downstream pathway of effective therapies, these lipids could serve as biomarkers for treatment response or even as direct treatment targets. However, it is important to stress that the cellular stress model utilized herein was based on a sustained 1 µM cortisol exposure over 4 days and therefore represents a supraphysiological glucocorticoid challenge rather than normal physiological cortisol dynamics. Accordingly, this model should be viewed primarily as an experimental system to elicit a robust stress-related lipidomic response, enabling qualitative comparison with the genetically implicated lipid signature, rather than as a quantitative mimic of in vivo cortisol exposure in depression. Thus, the overlap with MR results is most informative at the level of directional convergence and pathway involvement, not at the level of physiological cortisol dose equivalence.

Among the lipid species identified, cholesteryl ester 20:3 stands out as a potential key player. This lipid was supported by genetic evidence (MR and colocalization), showed changes in the experimental model (trending down with cortisol-induced cellular stress, up with Ze 117), and correlated with depressive symptoms in an independent cohort of young adults. CE(20:3) putatively contains dihomo-γ-linolenic acid (DGLA, 20:3 *n*-6) as its fatty acyl component—an intermediate in the omega-6 pathway between linoleic acid and arachidonic acid. Higher levels of CE(20:3) might reflect a greater availability of DGLA or a slower conversion of DGLA to arachidonic acid (perhaps due to reduced Δ5-desaturase activity). Intriguingly, DGLA itself has anti-inflammatory properties and can give rise to anti-inflammatory eicosanoids, which might contribute to its protective effect in depression [26,27]. Our observational finding that CE(20:3) is inversely related to depressive symptomatology (even in non-clinical individuals) reinforces its relevance. While causal inferences cannot be drawn from that correlation alone, it is consistent with the MR prediction. CE(20:3) could be further evaluated as a biomarker—for example, in epidemiological studies or as a predictor of antidepressant response. It also illustrates how narrowing down to the level of individual lipid species can provide leads (like DGLA metabolism) that would be obscured if one only looked at broad categories like total polyunsaturated fats.

The strengths of this work include the use of large genetic datasets to infer statistical causality, multiple layers of validation (sensitivity MR, replication in external cohorts, colocalization to verify shared variants), and integration of human genetics with human cell-based pharmacological experiments. Nevertheless, some limitations should be acknowledged. First, the MR approach assumes that the genetic instruments affect MDD only through their impact on the lipid exposure. While HEIDI filtering and MR-Egger intercept tests did not indicate widespread horizontal pleiotropy, we cannot rule out that some instruments have pleiotropic effects (e.g., via influencing another metabolite or trait) that could bias results. The colocalization analysis partly mitigates this concern by requiring a shared locus for lipid and MDD, but pleiotropy at a shared locus could still occur. Second, because MR inferences depend on the strength and composition of the genetic instruments (e.g., number of SNPs, *p*-value thresholds), true associations can be missed when instrument coverage is sparse. By leveraging multiple independent lipid GWAS panels as exposures, we substantially broadened the instrument set and thereby obtained a more comprehensive view of how the circulating lipidome relates to MDD. Third, our analyses were largely confined to individuals of European ancestry; thus, the findings may not generalize to other populations without further validation. Fourth, the PBMC cortisol model is a peripheral immune cell system and cannot capture the cellular complexity or region-specific lipid biology of the brain, which has unique lipid composition and dynamics [26,28]. Thus, the observed lipid changes should be interpreted as a peripheral stress-response signature rather than a direct model of neuronal or glial membrane remodeling. Relatedly, although peripheral lipids may reflect central processes to some extent, for example, via transport across the blood–brain barrier [29], and are therefore of interest as accessible biomarkers, blood–brain barrier transfer is not uniform across lipid classes: uptake is best established for free fatty acids and selected lysophospholipid species, whereas transport of many intact complex lipids remains uncertain and sterol transfer from blood to brain appears limited. Further studies are therefore required to establish whether the identified species, including CE(20:3), can qualify as accessible biomarkers. Fifth, given the exploratory and hypothesis-generating nature of this study, analyses were performed on lipid values normalized to internal standards without additional transformation in order to preserve sensitivity to large absolute intervention-related changes that might otherwise be attenuated. Accordingly, the findings are considered hypothesis-generating and require confirmation in independent studies. Sixth, while we demonstrated an overlap between genetic signals and an in vitro model, direct clinical evidence that modifying these lipids will alter depression risk is still needed. Randomized trials of pharmacological interventions targeting these lipid pathways would be a logical next step. Encouragingly, some nutritional trials with omega-3 supplements show a measurable, if modest, reduction in depression symptoms [23], and novel therapies that more selectively modulate lipid metabolism (such as inhibitors or activators of specific desaturase enzymes) could be explored in the future.

In conclusion, our findings reveal a statistically causal relationship between lipid metabolism and depression, characterized by a reduction in long-chain polyunsaturated lipid species. This lipidomic fingerprint—heavily influenced by the *FADS*-mediated desaturase pathway—provides a mechanistic link between genetic risk for MDD and known epidemiological factors such as diet and inflammation [26]. The observed convergence between genetically inferred lipidomic alterations, cortisol-induced membrane remodeling, and their partial normalization by an antidepressant (Ze 117) raises the possibility that stress-related lipid perturbations may represent an early, modifiable component of depression pathophysiology. Given that several of the implicated lipid species—such as CE(20:3) and long-chain phosphatidylcholines—were also associated with MDD risk in individuals without clinically verified depression, it appears plausible—without causality being established yet—that such lipid shifts precede symptom onset and contribute to disease vulnerability. Thus, a targeted modulation of lipid profiles under stress conditions might offer an opportunity for early intervention, potentially mitigating the transition from subclinical to manifest depressive illness. Prospective studies in at-risk populations, ideally incorporating longitudinal lipidomic profiling and stress markers, will be necessary to validate this hypothesis.

## 4. Materials and Methods

### 4.1. Mendelian Randomization

For lipidome exposures, we first relied on the most comprehensive lipidomic panel available from Cadby et al. [15]. This dataset includes GWAS summary statistics obtained from meta-analysis of blood samples, from *n* = 6057 individuals of European ancestry, spanning 578 lipid species (and 32 class totals). Additional lipid exposures were obtained from two independent studies: Ottensmann et al. (2023), which reported GWAS results for 179 plasma lipid species in *n* = 7174 Finnish individuals [17], and Harshfield et al. (2021), which reported GWAS results for 477 serum lipid traits (including many multilabel species) in *n* = 13,814 individuals of European ancestry (INTERVAL study) [18]. All GWAS summary statistics for these lipidomic studies (imputed SNP panels in the case of Cadby et al. and Ottensmann et al., genotyped SNPs in the case of Harshfield et al.) were downloaded from the NHGRI-EBI GWAS Catalog [30].

For broader metabolome context, we also analyzed GWAS summary statistics for 726 plasma metabolites measured on the Metabolon platform in *n* = 8455 European individuals (INTERVAL study, reported by [19]). These data were obtained from the OmicsPred repository (downloaded from https://www.omicspred.org/, accessed on 19 May 2025).

The outcome data for depression were obtained from the Psychiatric Genomics Consortium (PGC). We used the largest available GWAS of MDD (excluding 23andMe participants for data availability reasons), comprising 412,305 cases and 1,588,397 controls of primarily European ancestry [16]. This meta-analysis included broad (“extended”) definitions of depression (e.g., self-reported and clinically diagnosed cases). To evaluate clinical relevance, we also obtained a subset GWAS of clinically ascertained MDD cases (major depressive disorder diagnosed via clinical interview) from the same study (26,778 cases, 51,857 controls). For comparison with another mood-related disorder, we retrieved GWAS summary statistics for bipolar disorder (European ancestry, excluding 23andMe), recently published by O’Connell et al. (2025) [21].

We performed MR analyses using generalized summary-data-based Mendelian randomization (GSMR), which leverages multiple, moderately correlated SNP instruments per exposure while accounting for horizontal pleiotropy [31,32]. For each lipid (or metabolite) exposure, we selected SNP instruments meeting a relaxed association threshold of *p* < 1 × 10^−6^ (as opposed to the conventional genome-wide significance of 5 × 10^−8^) to increase the number of instruments and traits testable. SNPs overlapping between exposure and outcome GWASs were retained and harmonized. Palindromic SNPs were excluded, and markers were clumped for linkage disequilibrium (LD) at r^2^ < 0.1 within a 10 Mb window. We then applied the GSMR method (GSMR v2.0) excluding remaining correlated markers (threshold: r^2^ < 0.1) and pleiotropic SNPs with HEIDI outlier filtering (HEIDI *p* < 0.01). LD correlations were estimated from the 1000 Genomes European reference panel. In the primary analysis, we required a minimum of 10 SNP instruments per lipid; this yielded 187 lipid species in the Cadby et al. discovery panel that were tested for causal association with MDD. Multiple-testing correction was performed using the Benjamini–Hochberg procedure across these tests. For completeness, we also calculated GSMR estimates for species that had at least 3 SNP instruments (relaxing the instrument count criterion); these results are reported in the Supplementary Materials. Sensitivity analyses for significant hits were performed using the GSMR-selected SNP instruments under alternative MR methods—specifically inverse-variance weighting (IVW) and MR-Egger regression—as implemented in the MendelianRandomization R package [33]. These methods provide additional robustness against pleiotropy, with MR-Egger intercepts used to test for directional pleiotropy.

Mapping of lipid species across studies (e.g., between different GWAS panels or between GWAS and experimental data) was done using the RefMet metabolite nomenclature database [34], ensuring consistent identification of species by their molecular composition.

### 4.2. Colocalization Analysis

To complement the MR results, we carried out Bayesian colocalization analysis for each lipid trait and MDD. We applied this analysis to all lipidomic and metabolomic datasets for which imputed SNP-level GWAS summary statistics were available—namely, the panels from Cadby et al., Ottensmann et al., and Xu et al. For a given lipid–MDD pair, we first defined genomic regions around each index SNP associated with the lipid (*p* < 1 × 10^−6^, clumped at LD r^2^ < 0.1 within 500 kb). Within each such region (±250 kb around the index SNP), we computed the posterior probability of a shared causal variant (colocalization probability PPH4) using the coloc R package’s default (approximate Bayes factor, *abf*) method [35]. Regions with strong evidence for colocalization (PPH4 > 0.8) were recorded, and those with moderate support (PPH4 > 0.5) were noted as suggestive. Overlapping colocalized regions (either overlapping in genomic position or implicating the same locus across multiple lipids) were merged into single loci for interpretability. For each colocalized locus, we identified candidate causal variants by extracting either variants with SNP PPH4 > 0.01 or those in the 95% credible set and mapped these variants to their nearest protein-coding genes (using GENCODE v47 on GRCh37). Regional association plots were generated for key loci using LocusZoom [36].

### 4.3. PBMC Lipid Perturbation Study

We leveraged data from a recent in vitro study of lipid changes under cortisol and Ze 117 treatment in peripheral blood mononuclear cells (PBMCs; see Bussmann et al. (2023) [13]). Ze 117 is a proprietary St. John’s wort extract characterized by a low hyperforin content (≤0.2%), which distinguishes it pharmacologically from other St. John’s wort extracts. Details of this experimental model and lipidomics data were previously described by Bussmann et al. (2023) [13]. In brief, human PBMCs were exposed to 1 µM cortisol for 4 days to mimic stress, with or without co-treatment with Ze 117 (St. John’s wort extract) at 10, 25, and 50 µg/mL. The cellular lipidome was profiled by mass spectrometry by Lipotype GmbH (Dresden, Germany) and the identified lipid molecules were quantified by normalization to a specific internal standard using Lipotype’s in-house data management system. One experimental batch deviating from the other samples on principal components analysis was excluded from downstream analysis, yielding 20 samples remaining for analysis [13]. For our analysis, we excluded lipid features with >50% missing values across samples, yielding 500 measured lipid features (out of 890 initial features). Missing values in the remaining features were imputed as zero (reflecting levels below detection). Differential lipid abundance on absolute quantification (pmol) between conditions was assessed using linear models with treatment contrasts of interest (cortisol vs. control and cortisol+Ze 117 50 µg vs. cortisol alone), accounting for experimental batch as a covariate. These models were implemented with the lsmeans package in R [37]. Benjamini–Hochberg false discovery rate correction was applied separately for each contrast.

To compare the PBMC findings with the genetic results, we mapped the PBMC lipid features to the GWAS lipid species (when possible) using the RefMet database [34]. Overlap analysis of GSMR hits among species differentially regulated upon cortisol administration or co-incubation with Ze 117 was performed by randomly shuffling the PBMC conditions within each batch, prior to repeating the linear model analysis across all features: the top N features were retained, with N corresponding to the number of species altered under cortisol or Ze 117 administration (132 and 238 respectively), and mapped to the GWAS lipids panel. Empirical *p*-value was hence determined by the number of shuffling occurrences yielding a number of GSMR hits equal or higher to the number observed with experimental data, over 1000 repeats. This procedure ensures that the overlap tests account for the correlation structure inherent to the PBMC lipid panel.

### 4.4. Correlation Analysis Between Depressive Symptoms and CE(20:3) Plasma Levels

A total of *n* = 970 healthy young adults (mean age: 22 y; 64% females) completed the Montgomery–Åsberg Depression Rating Scale (MADRS) [38,39]. Lipidomic profiles were obtained from plasma samples using the Metabolon Complex Lipids Platform: lipids were extracted from samples in methanol:dichloromethane in the presence of internal standards. The extracts were concentrated under nitrogen and reconstituted in 0.25 mL of 10 mM ammonium acetate dichloromethane:methanol (50:50). The extracts were transferred to inserts and placed in vials for infusion-MS analysis, performed on a Shimadzu LC with nano PEEK tubing and the Sciex SelexIon-5500 QTRAP. The samples were analyzed via both positive and negative mode electrospray. The 5500 QTRAP scan was performed in MRM mode with a total of more than 1100 MRMs. Individual lipid species were quantified by taking the peak area ratios of target compounds and their assigned internal standards, then multiplying by the concentration of internal standard added to the sample.

Prior to correlation analysis, CE(20:3) levels were trimmed for outliers (exclusion of values lying outside mean ± 5 s.d. range), log transformed and adjusted for covariates using linear regression (age at blood sampling, sex, recruitment center, lipidomics batch and body mass index). Depressive symptoms, modeled as the total score across the ten MADRS items, were adjusted for age, sex and recruiting wave using linear regression. Association between CE(20:3) levels and depressive symptoms was assessed using Pearson’s correlation.

## Data Availability

The data that support the findings of this study are available from the corresponding author upon request. Data are located in controlled-access data storage at the University of Basel.

## Supplementary Materials

The following supporting information can be downloaded at: https://www.mdpi.com/article/10.3390/ijms27104344/s1.

## Abbreviations

The following abbreviations are used in this manuscript:

abf	approximate Bayes factor (coloc method)
BD	bipolar disorder
BMI	body mass index
CE	cholesteryl ester (e.g., CE(20:3))
DGLA	dihomo-γ-linolenic acid (20:3 *n*-6)
DG	diacylglycerol (e.g., DG(18:1/20:3))
FADS	fatty acid desaturase gene cluster (FADS1/2)
FDR	false discovery rate
GC/GPC/GPE	glycerophosphocholine/glycerophosphatidylcholine (often written as GPC)/glycerophosphatidylethanolamine (often written as GPE)
GSMR	generalized summary-data-based Mendelian randomization
GWAS	genome-wide association study
HEIDI	heterogeneity in dependent instruments (outlier filtering in GSMR)
IVW	inverse-variance weighting (MR method)
LC	liquid chromatography
LD	linkage disequilibrium
LPC	lysophosphatidylcholine
LPE	lysophosphatidylethanolamine
MADRS	Montgomery–Åsberg Depression Rating Scale
MDD	major depressive disorder
MR	Mendelian randomization
MRM	multiple reaction monitoring (mass spectrometry acquisition mode)
MS	mass spectrometry
PBMC	peripheral blood mononuclear cell(s)
PC	phosphatidylcholine (includes plasmalogen forms such as PC(O-...) and PC(P-...))
PGC	Psychiatric Genomics Consortium
PI	phosphatidylinositol
PPH4	posterior probability of a shared causal variant (colocalization metric)
PUFA	polyunsaturated fatty acid(s)
RefMet	reference metabolite nomenclature database
SNP	single-nucleotide polymorphism
SM	sphingomyelin
SSRI	selective serotonin reuptake inhibitor
Ze 117	St. John’s wort extract (Hypericum perforatum extract; “Ze 117”)
