# Supplementary material for: Mendelian Randomization Identifies Lipidomic Signatures of Depression Risk That Are Partly Reflected in Cortisol-Induced Membrane Remodeling and Modulated by St. John’s Wort Extract (Ze 117)
Source: Int J Mol Sci. 2026 May 13;27(10):4344. doi: 10.3390/ijms27104344 (PMC13207717; doi:10.3390/ijms27104344)

Figure S1: Mendelian Randomization estimates across different methods.

List of 49 individual lipid species showing significant GSMR estimates (FDR <0.05) with MDD. x-axis: MR estimates (b_{xy}) provided for GSMR, IVW and Egger methods. Error bars correspond to 95% confidence intervals.

CE: Cholesteryl Ester; DAG: Diacylglycerol; LPC: Lysophosphatidylcholine; LPE: Lysophosphatidylethanolamine; PC: Phosphatidylcholine; PE: Phosphatidylethanolamine; PI: Phosphatidylinositol ; SM: Sphingomyelin; TAG: Triacyglycerol; SP: Sphingolipids.

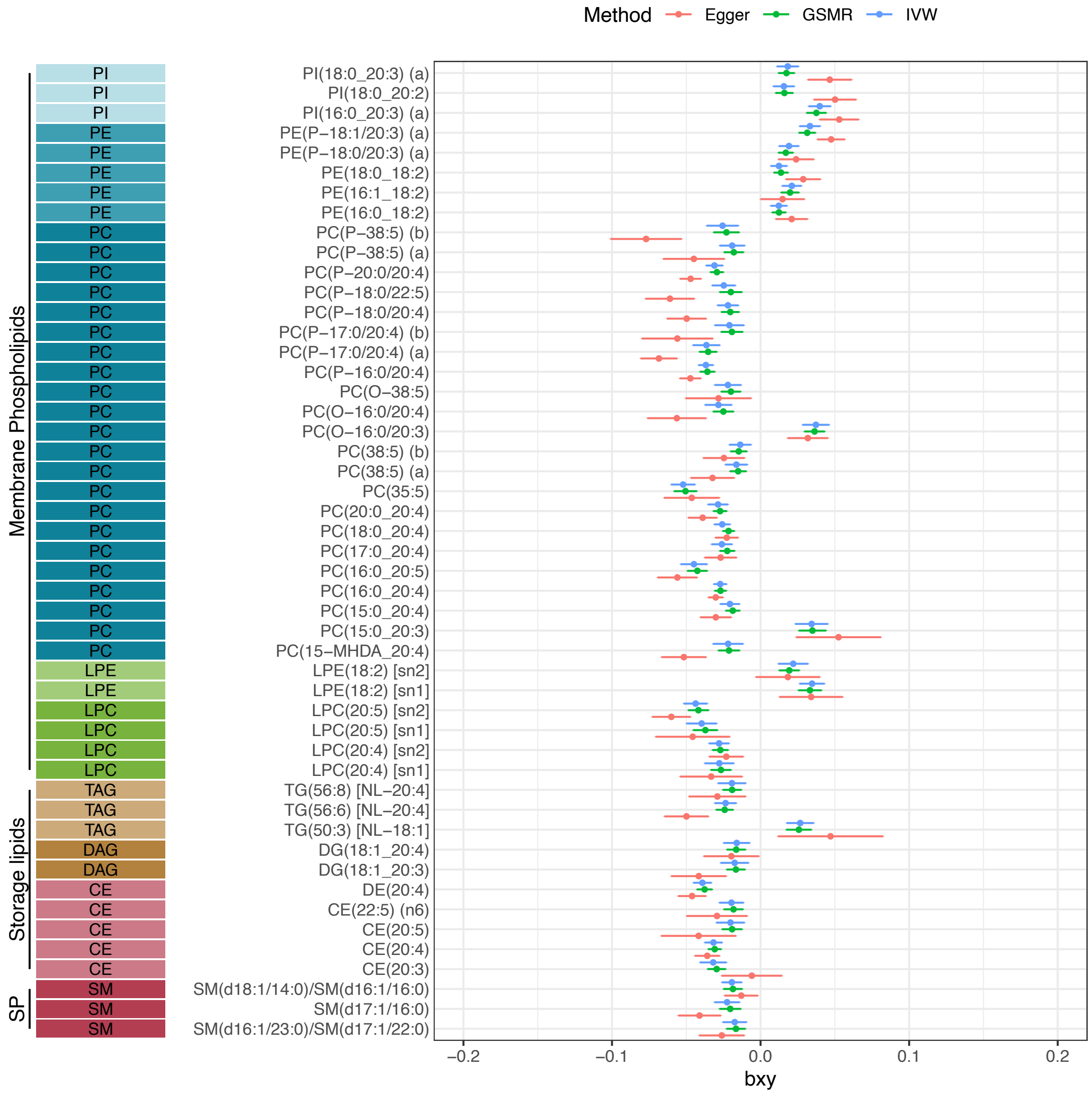

Figure S2: Results of GSMR analysis between lipids species and clinical MDD.

Shown are 26 species showing significant GSMR estimates (FDR <0.05) with MDD GWAS restricted to clinical/interview based phenotypes. Error bars represent 95% confidence intervals. CE: Cholesteryl Ester; DAG: Diacylglycerol; LPC: Lysophosphatidylcholine; LPE: Lysophosphatidylethanolamine; PC: Phosphatidylcholine; PE: Phosphatidylethanolamine; PI: Phosphatidylinositol ; HexCer: Hexosylceramide; TAG: Triacyglycerol; SP: Sphingolipids.

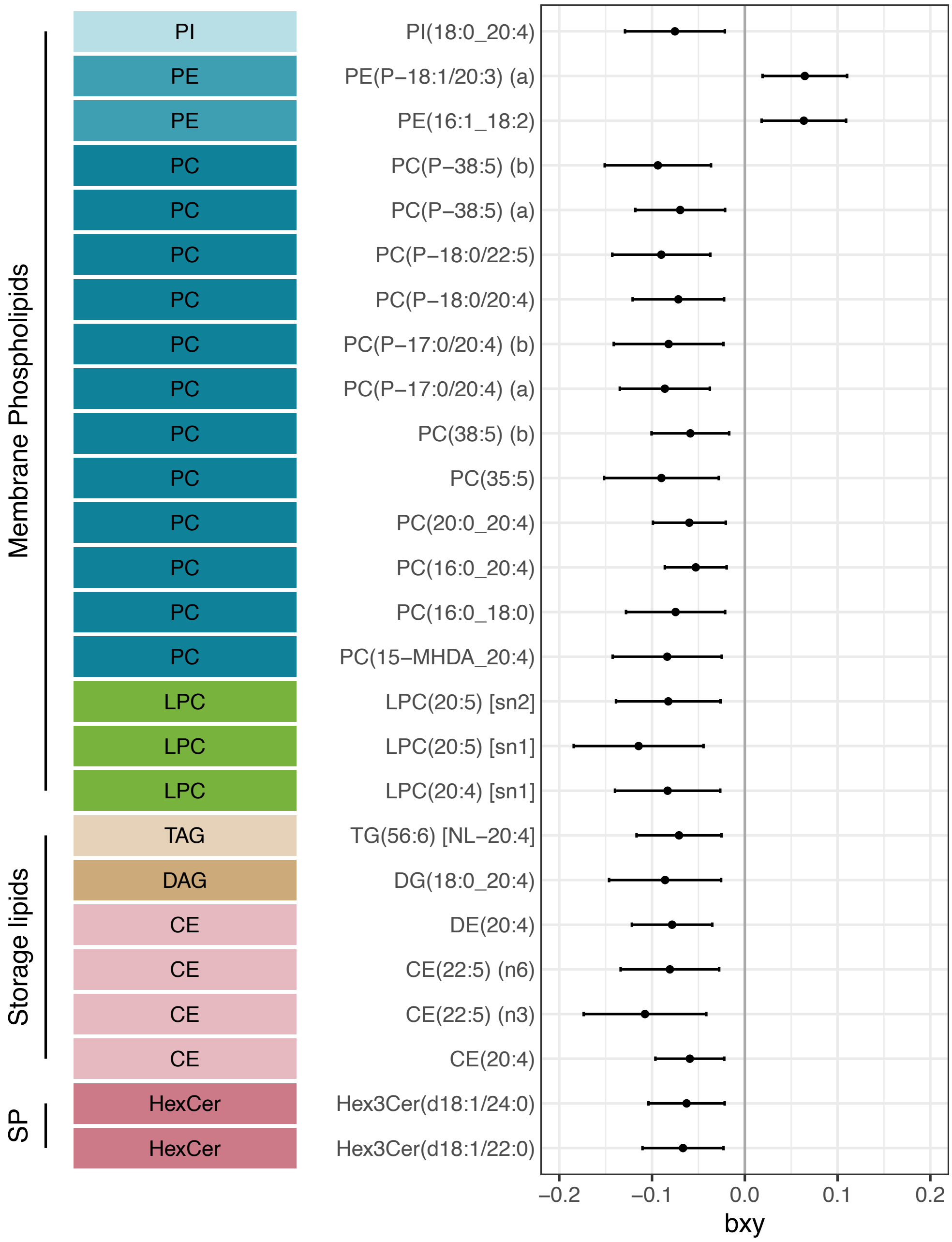

Figure S3: Results of GSMR analysis for MDD in two additional lipidomics studies.

A: List of 18 individual lipid species from Ottensmann et al. showing significant GSMR estimates (FDR <0.05) with MDD.
B: List of 14 individual lipid measures from Harshfield et al. showing significant GSMR estimates (FDR < 0.05) with MDD.
Error bars correspond to 95% confidence intervals.
CE: Cholesteryl Ester; DAG: Diacylglycerol; LPC: Lysophosphatidylcholine; LPE: Lysophosphatidylethanolamine; PC: Phosphatidylcholine; PE: Phosphatidylethanolamine; PG: Phosphoglycerol; PI: Phosphatidylinositol ; SM: Sphingomyelin; SP: Sphingolipids.

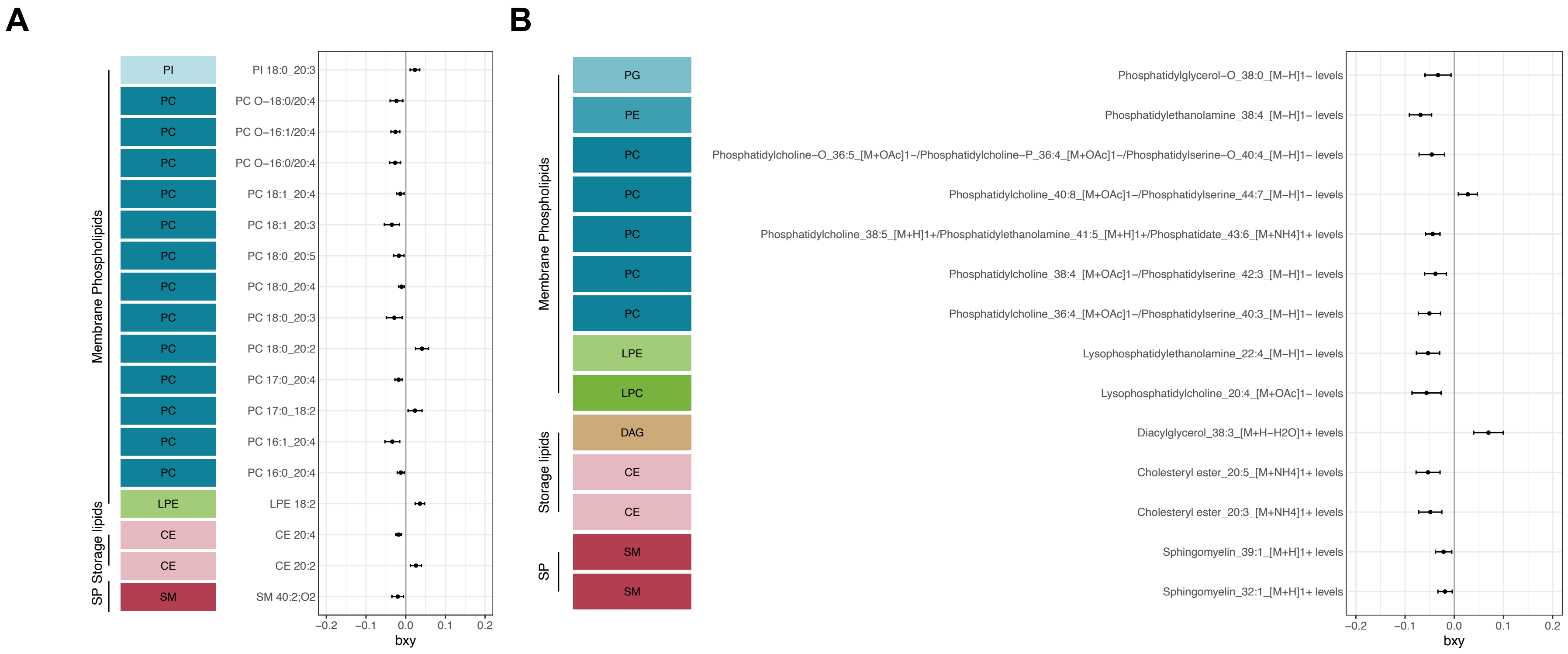

Figure S4: Evaluation of Cadby et al. GSMR hits for MDD in two additional lipidomics studies.

Horizontal axis represents GSMR estimates of the 49 hits from Cadby et al. study overlapping with Ottensmann et al. study species (Left) or Harshfield et al. species (Right).
Vertical axis represents GSMR estimate in the respective studies.
Species showing nominal association (*p* <.05) with consistent direction of effect in additional studies are highlighted in blue.
CE: Cholesteryl Ester; LPC: Lysophosphatidylcholine; LPE: Lysophosphatidylethanolamine; PC: Phosphatidylcholine; PE: Phosphatidylethanolamine; PI: Phosphatidylinositol; TG: Triacyglycerol

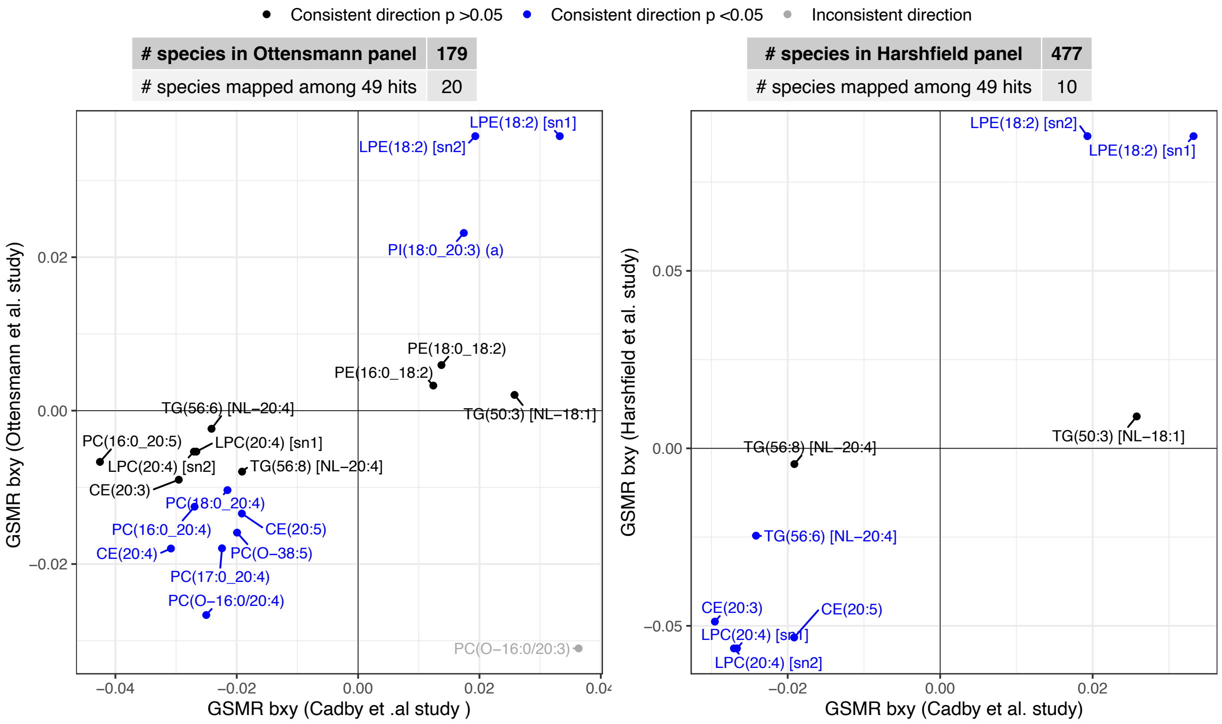

Figure S5: Results of GSMR analysis between plasma metabolites and MDD.

Shown are 38 metabolites showing significant GSMR estimates (FDR<0.05) with MDD. Error bars represent 95% confidence intervals.

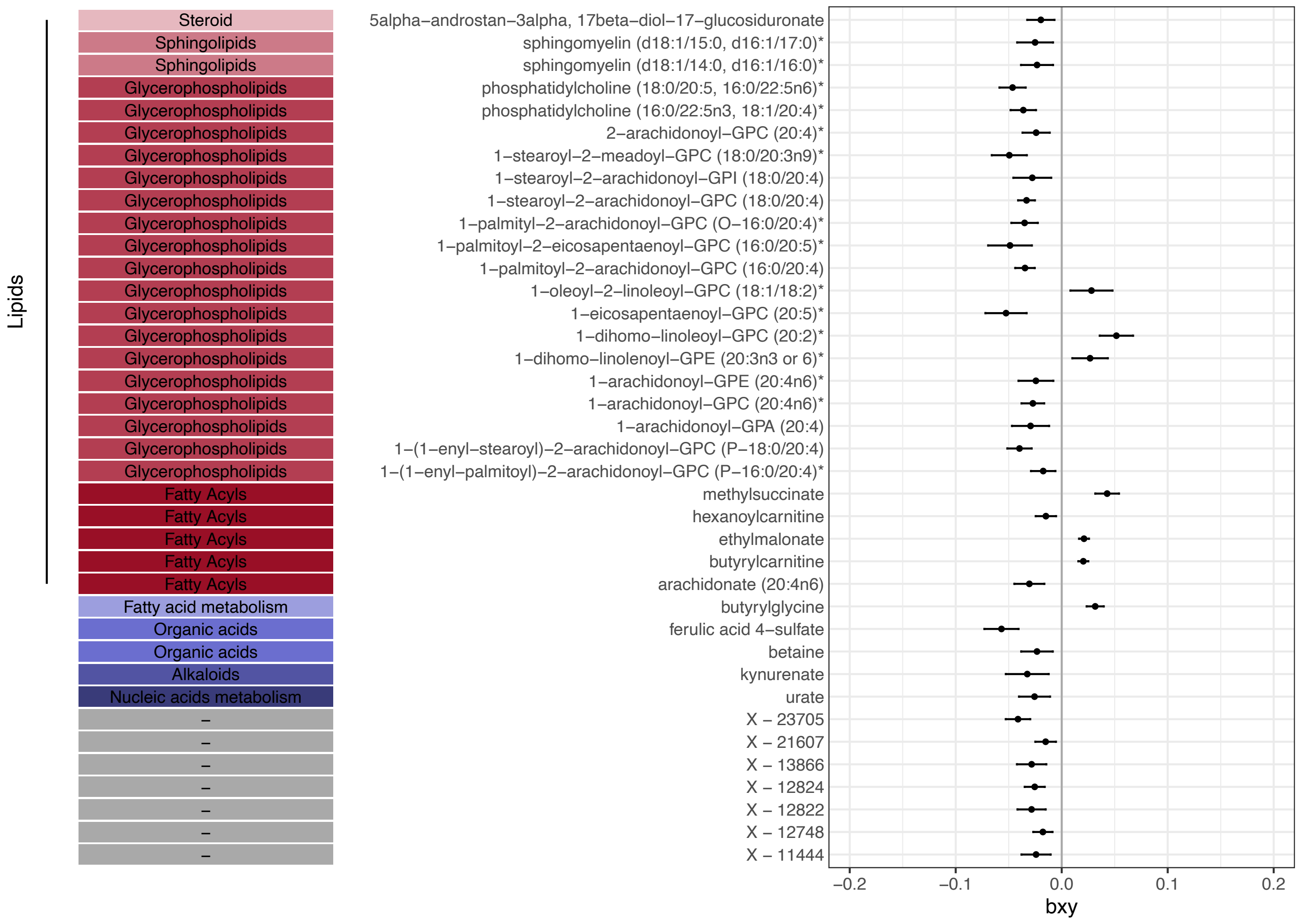

Figure S6: Results of GSMR analysis between lipids or metabolites and MDD with and without inclusion of the FADS genes cluster.

For each study, the GSMR estimates obtained under inclusion (x-axis) or exclusion (y-axis) of the *FADS* genomic locus on chromosome 11 were compared. Species showing significant association in the initial analysis (*FADS* locus inclusion) are highlighted in yellow. Species also showing nominal association under *FADS* locus exclusion are labeled.

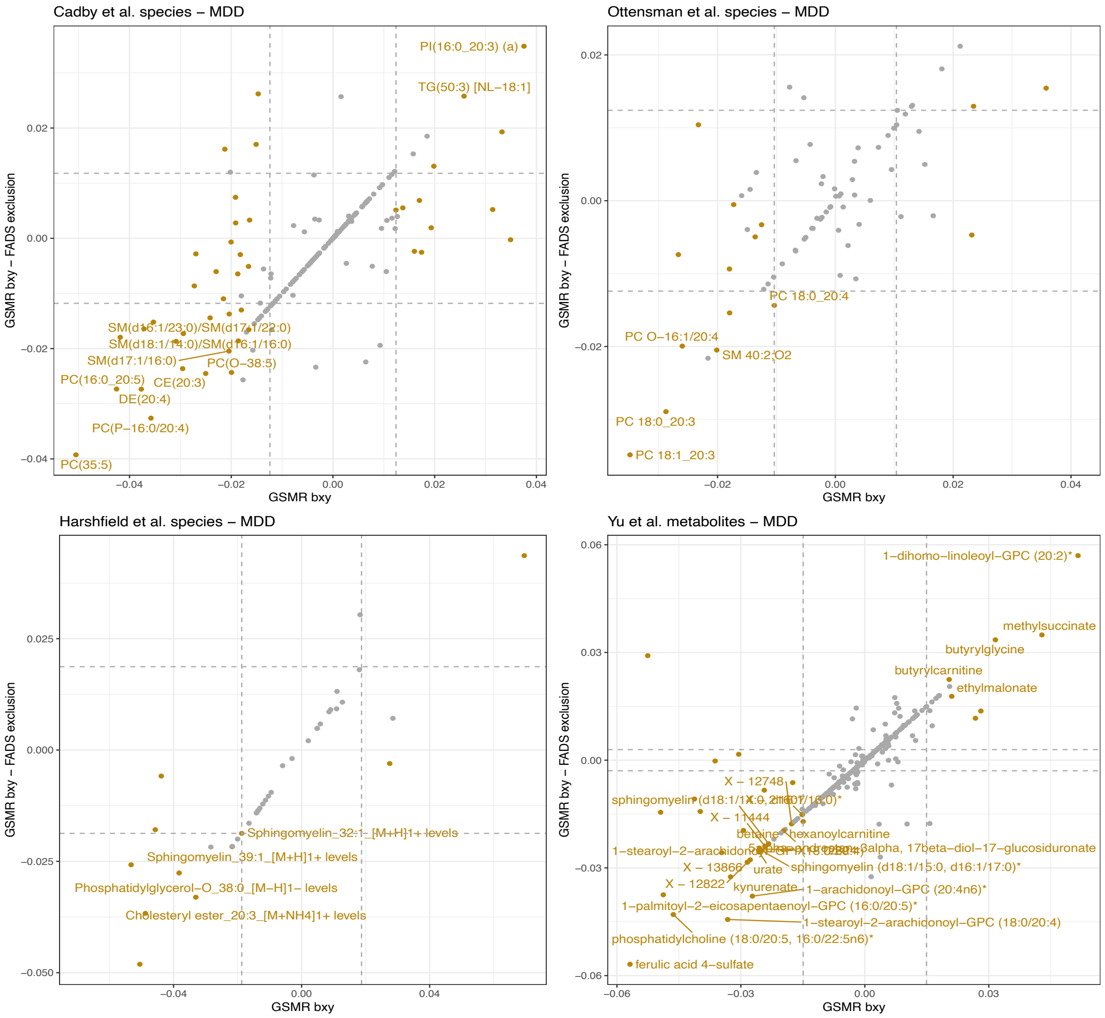

Figure S7: Comparison of GSMR estimates of lipids species on MDD and BIP.

Each dot represents one lipid species from Cadby et al. lipidomic study.
Horizontal axis: GSMR estimate for MDD. Vertical axis: GSMR estimate for BIP.
Species showing significant association (FDR<.05) for both outcomes are highlighted in yellow. Species showing significant association for MDD and nominal p > 0.05 for BIP are highlighted in magenta. Species showing significant association for BIP and nominal p > 0.05 for MDD are highlighted in blue.

CE: Cholesteryl Ester; DG: Diacylglycerol; PC: Phosphatidylcholine; PE: Phosphatidylethanolamine; PG: Phosphoglycerol; SM: Sphingomyelin; TG: Triacyglycerol.

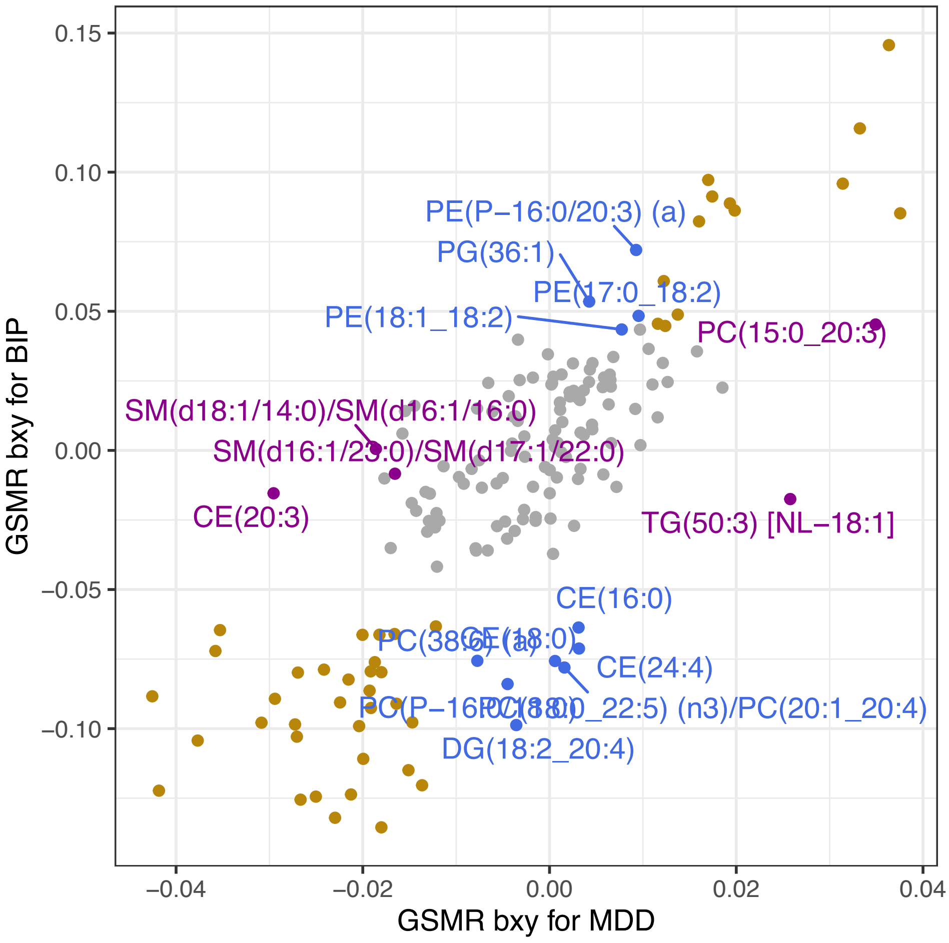

Figure S8: Distribution of MADRS Total score for depressive symptoms in N=970 healthy young adults and association between CE(20:3) plasma levels and MADRS score in this population.

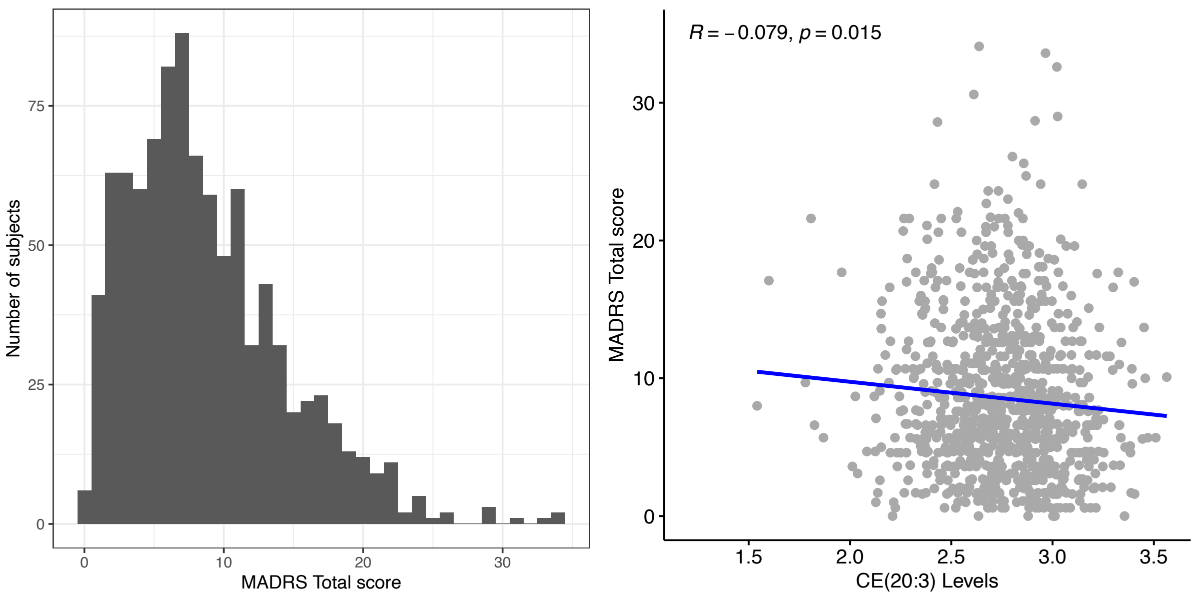

Supplement: Supplementary file 1 [file ijms-27-04344-s001.zip › Supplementary_Figures_1-8_revision_final.pdf]
